# Collagen triple helix repeat containing-1 promotes functional recovery of sweat glands by inducing adjacent microvascular network reconstruction *in vivo*

**DOI:** 10.1093/burnst/tkac035

**Published:** 2022-08-02

**Authors:** Xingyu Yuan, Xianlan Duan, Zhao Li, Bin Yao, Enhejirigala, Wei Song, Yi Kong, Yuzhen Wang, Fanliang Zhang, Liting Liang, Shijun Zhu, Mengde Zhang, Chao Zhang, Sha Huang, Xiaobing Fu

**Affiliations:** School of Medicine, Nankai University, 94 Wei Jin Road, Tianjin 300071, PR China; Research Center for Tissue Repair and Regeneration affiliated to the Medical Innovation Research Department, PLA General Hospital, 28 Fu Xing Road, Beijing 100853, PR China; School of Medicine, Nankai University, 94 Wei Jin Road, Tianjin 300071, PR China; Research Center for Tissue Repair and Regeneration affiliated to the Medical Innovation Research Department, PLA General Hospital, 28 Fu Xing Road, Beijing 100853, PR China; Research Center for Tissue Repair and Regeneration affiliated to the Medical Innovation Research Department, PLA General Hospital, 28 Fu Xing Road, Beijing 100853, PR China; PLA Key Laboratory of Tissue Repair and Regenerative Medicine and Beijing Key Research Laboratory of Skin Injury, Repair and Regeneration, Chinese PLA General Hospital and PLA Medical College, 51 Fu Cheng Road, Beijing 100048, PR China; Research Center for Tissue Repair and Regeneration affiliated to the Medical Innovation Research Department, PLA General Hospital, 28 Fu Xing Road, Beijing 100853, PR China; PLA Key Laboratory of Tissue Repair and Regenerative Medicine and Beijing Key Research Laboratory of Skin Injury, Repair and Regeneration, Chinese PLA General Hospital and PLA Medical College, 51 Fu Cheng Road, Beijing 100048, PR China; Academy of Medical Engineering and Translational Medicine, Tianjin University, 92 Weijin Road, Tianjin, 300072, PR China; Research Center for Tissue Repair and Regeneration affiliated to the Medical Innovation Research Department, PLA General Hospital, 28 Fu Xing Road, Beijing 100853, PR China; College of Graduate, Tianjin Medical University, Tianjin 300070, PR China; Institute of Basic Medical Research, Inner Mongolia Medical University, Hohhot 010110, Inner Mongolia, PR China; Research Center for Tissue Repair and Regeneration affiliated to the Medical Innovation Research Department, PLA General Hospital, 28 Fu Xing Road, Beijing 100853, PR China; PLA Key Laboratory of Tissue Repair and Regenerative Medicine and Beijing Key Research Laboratory of Skin Injury, Repair and Regeneration, Chinese PLA General Hospital and PLA Medical College, 51 Fu Cheng Road, Beijing 100048, PR China; Research Center for Tissue Repair and Regeneration affiliated to the Medical Innovation Research Department, PLA General Hospital, 28 Fu Xing Road, Beijing 100853, PR China; PLA Key Laboratory of Tissue Repair and Regenerative Medicine and Beijing Key Research Laboratory of Skin Injury, Repair and Regeneration, Chinese PLA General Hospital and PLA Medical College, 51 Fu Cheng Road, Beijing 100048, PR China; Research Center for Tissue Repair and Regeneration affiliated to the Medical Innovation Research Department, PLA General Hospital, 28 Fu Xing Road, Beijing 100853, PR China; PLA Key Laboratory of Tissue Repair and Regenerative Medicine and Beijing Key Research Laboratory of Skin Injury, Repair and Regeneration, Chinese PLA General Hospital and PLA Medical College, 51 Fu Cheng Road, Beijing 100048, PR China; Department of Burn and Plastic Surgery, Air Force Hospital of Chinese PLA Central Theater Command, Datong 037000, Shanxi, PR China; Research Center for Tissue Repair and Regeneration affiliated to the Medical Innovation Research Department, PLA General Hospital, 28 Fu Xing Road, Beijing 100853, PR China; PLA Key Laboratory of Tissue Repair and Regenerative Medicine and Beijing Key Research Laboratory of Skin Injury, Repair and Regeneration, Chinese PLA General Hospital and PLA Medical College, 51 Fu Cheng Road, Beijing 100048, PR China; Research Center for Tissue Repair and Regeneration affiliated to the Medical Innovation Research Department, PLA General Hospital, 28 Fu Xing Road, Beijing 100853, PR China; School of Medicine, Nankai University, 94 Wei Jin Road, Tianjin 300071, PR China; Research Center for Tissue Repair and Regeneration affiliated to the Medical Innovation Research Department, PLA General Hospital, 28 Fu Xing Road, Beijing 100853, PR China; Research Center for Tissue Repair and Regeneration affiliated to the Medical Innovation Research Department, PLA General Hospital, 28 Fu Xing Road, Beijing 100853, PR China; PLA Key Laboratory of Tissue Repair and Regenerative Medicine and Beijing Key Research Laboratory of Skin Injury, Repair and Regeneration, Chinese PLA General Hospital and PLA Medical College, 51 Fu Cheng Road, Beijing 100048, PR China; School of Medicine, Nankai University, 94 Wei Jin Road, Tianjin 300071, PR China; Research Center for Tissue Repair and Regeneration affiliated to the Medical Innovation Research Department, PLA General Hospital, 28 Fu Xing Road, Beijing 100853, PR China; Research Center for Tissue Repair and Regeneration affiliated to the Medical Innovation Research Department, PLA General Hospital, 28 Fu Xing Road, Beijing 100853, PR China; School of Medicine, Nankai University, 94 Wei Jin Road, Tianjin 300071, PR China; Research Center for Tissue Repair and Regeneration affiliated to the Medical Innovation Research Department, PLA General Hospital, 28 Fu Xing Road, Beijing 100853, PR China; PLA Key Laboratory of Tissue Repair and Regenerative Medicine and Beijing Key Research Laboratory of Skin Injury, Repair and Regeneration, Chinese PLA General Hospital and PLA Medical College, 51 Fu Cheng Road, Beijing 100048, PR China; Research Unit of Trauma Care, Tissue Repair and Regeneration, Chinese Academy of Medical Sciences, 2019RU051, Beijing 100048, PR China

**Keywords:** Collagen triple helix repeat containing-1, Sweat glands, Microvascular network, Dermal microvascular endothelial cells, Reconstruction

## Abstract

**Background:**

Sweat glands (SGs) have low regenerative potential after severe burns or trauma and their regeneration or functional recovery still faces many obstacles. In practice, restoring SG function requires not only the structural integrity of the gland itself, but also its neighboring tissues, especially blood vessels. Collagen triple helix repeat containing-1 (CTHRC1) was first identified in vascular repair, and increasing reports showed a close correlation between cutaneous appendage specification, patterning and regeneration. The purpose of the present study was to clarify the role of CTHRC1 in SGs and their adjacent microvessels and find therapeutic strategies to restore SG function.

**Methods:**

The SGs and their adjacent microvascular network of *Cthrc1*^−/−^ mice were first investigated using sweat test, laser Doppler imaging, tissue clearing technique and transcriptome analysis. The effects of CTHRC1 on dermal microvascular endothelial cells (DMECs) were further explored with cell proliferation, DiI-labeled acetylated low-density lipoprotein uptake, tube formation and intercellular junction establishment assays. The effects of CTHRC1 on SG function restoration were finally confirmed by replenishing the protein into the paws of *Cthrc1*^−/−^ mice.

**Results:**

CTHRC1 is a key regulator of SG function in mice. At the tissue level, *Cthrc1* deletion resulted in the disorder and reduction of the microvascular network around SGs. At the molecular level, the knockout of *Cthrc1* reduced the expression of vascular development genes and functional proteins in the dermal tissues. Furthermore, CTHRC1 administration considerably enhanced SG function by inducing adjacent vascular network reconstruction.

**Conclusions:**

CTHRC1 promotes the development, morphogenesis and function execution of SGs and their neighboring vasculature. Our study provides a novel target for the restoration or regeneration of SG function *in vivo*.

Highlights

- CTHRC1 is a key regulator of SG function in mice.
- CTHRC1 deficiency had a few effects on SG morphogenesis, but resulted in the disorder and reduction of the microvascular network around SGs.
- Knockout of *Cthrc1* had no effect on the expression of SG marker genes and proteins but reduced the expression of vascular development genes and functional proteins in the dermal tissues.
- CTHRC1 administration considerably enhanced SG function by improving the angiogenic capacity of dermal microvascular endothelial cells and inducing adjacent vascular network remodeling.

## Background

Sweating is an essential physiological process in thermoregulation through water evaporation during sports or in extreme climates [1, 2]. Insufficient or no sweating (i.e. hypohidrosis and anhidrosis) causes hyperthermia, exhaustion, shock and possibly death [3, 4]. In contrast, excessive sweating (i.e. hyperhidrosis) generates dehydration, dermatopathy or social discomfort [5]. Sweating is the primary function of sweat glands (SGs), and the execution of this process requires precise coordination of the secretory coils, ducts and the adjacent microvascular and nervous systems [6–8]. However, the skin and its appendages (i.e. SGs) are often partially or entirely lost in severe burns or trauma [9, 10] and their regeneration or functional restoration during clinical treatment still faces many challenges [6, 8, 11, 12].

Collagen triple helix repeat containing-1 (CTHRC1), a secreted extracellular matrix (ECM) protein, was first identified in adventitial fibroblasts and neointimal smooth muscle cells of injured arties [13]. It is known to be mainly involved in vascular remodeling and angiogenesis by promoting cell migration and limiting collagen synthesis [14–17]. In clinical studies, mutations in this gene are mainly associated with Barrett’s esophagus and esophageal adenocarcinoma [18]. In contrast, high expression of CTHRC1 is related to angiogenesis in lung adenocarcinoma [16]. For the past few years, an increasing number of studies have reported a close correlation between the expression of CTHRC1 and wound healing, skin appendage specification, patterning and regeneration. An analysis of acute wound healing mouse models found that CTHRC1 accelerated wound repair by increasing the M2 macrophage population and transforming growth factor beta (TGF-β) expression level [19]. The anterior–posterior polarity of mouse hair follicles (HFs) (referred to as HF arrangement) is regulated by the planar cell polarity. Another study reported that CTHRC1 could act as a downstream effector of frizzled 6 to enhance Wnt/planar cell polarity signaling and ultimately regulate the orientation of HFs [20]. Moreover, a further paper revealed that the deletion of *Cthrc1* shortened the length of the HF bud invagination in fetal mice, reduced HF diameter in adult mice, and even decreased HF regeneration capacity [9]. Recently, our findings showed that CTHRC1 was a crucial biochemical regulator, and it could cooperate with heme oxygenase 1 (HMOX1) to direct the conversion of mesenchymal stem cells into functional SGs in a 3D bioprinted matrix [21]. However, although we verified that CTHRC1 is a critical protein in the microenvironment for SG differentiation *in vitro*, its biological role in the development, morphogenesis and function execution of SGs and their neighboring tissues *in vivo* is still unknown.

The anatomy of SGs and surrounding tissues were gradually delineated by histological analysis from the 1950s [7, 22]. SGs are unbranched coiled tubules comprising mainly intraepidermal ducts, straight ducts and secretory coils [8]. The overall tissue architecture of SGs is a bilayered tube structure with secretory cells as the inner layer and myoepithelial cells encapsulated by the basement membrane as the outer layer [1]. Blood vessels are one of the neighboring vital tissues of SGs and are considered to support the reabsorption of sweat components [8]. The 3D anatomy of blood vessels revealed that these capillaries were intertwined with or ran alongside SG tubules similar to peritubular capillaries and proximal renal tubules in the kidney [7, 23]. In practice, the restoration of SG function requires not only its structural integrity but also the support of peripheral blood vessels. An exploratory study recently indicated that a vascularized SG structure was obtained by transplanting Matrigel-embedded SG cells under the inguinal skin of mice, thus providing reference information for restoring or regenerating SG function *in vivo* [6].

In this study, we initially determined the role of CTHRC1 in SG function using sweat test, laser Doppler imaging and 3D reconstruction of SGs and their adjacent microvascular network in wild type (WT) and *Cthrc1*^−/−^ mice. Then, we confirmed the effect of *Cthrc1* deletion on the expression of vascular development genes and functional proteins with transcriptome and co-localization analysis. Finally, we identified CTHRC1 as a potential key regulator of the impaired angiogenic ability of *Cthrc1*^−/−^ dermal microvascular endothelial cells (DMECs) and found that CTHRC1 administration promoted the recovery of SG function by inducing adjacent microvascular network reconstruction. Our work provides a novel target for SG function repair and broadens our horizon with regard to the regeneration of vascularized organs.

## Methods

### Animals

C57BL/6 mice (6–8 weeks of age) were purchased from SPF Biotechnology Co., Ltd. Whole-body *Cthrc1*^−/−^ mice (Riken BioResource Center, Acc. No: RBRC03519) were generously gifted by Prof. Zhigang Zhang (Ren Ji Hospital, Shanghai Jiao Tong University). Animals were maintained in a specific-pathogen-free facility of the Chinese PLA General Hospital at 23 ± 3°C with a 12/12-light/dark cycle. All animal experiments were approved by the Institutional Animal Care and Use Committee of the Chinese PLA General Hospital (Beijing, China).

### Sweat test

Iodine/ethanol (2% w/v) and starch/castor oil solution (1 g/ml) were applied to the hind paw pads of anesthetized mice at 5-min intervals. Afterward, 50 μl of 100 μM acetylcholine (Sigma-Aldrich, USA) was subcutaneously injected into the hind paws. Sweat dots appeared after 10 min and were photographed immediately.

### Light-sheet microscopy

The anatomical structure of SGs and their surrounding microvascular networks were visualized using the PEGASOS tissue clearing protocol as previously described [24]. Briefly, the hind feet were first isolated from the anesthetized mice after tail vein injection of *Lycopersicon Esculentum* lectin (500 μg/ml; Thermofisher, L32472) and cardiac perfusion. Then, the samples were washed with phosphate-buffered saline (PBS) and fixed with 4% paraformaldehyde (Solarbio, China) at 4°C for 24 h. Finally, the PEGASOS method was carried out to clear the samples for 7 days and they were immediately imaged in the light-sheet imaging chamber. The light-sheet microscopy images of SGs and their adjacent microvascular networks were visualized by autofluorescence (excitation/emission: 480/520 nm) and Dylight 649-conjugated lectin (excitation/emission: 655/670 nm), respectively. All specimens were imaged with Zeiss Z.1 Light-sheet microscope (Germany). 3D images and movies were generated using the ‘volume rendering’ and ‘animation’ function of Imaris 9.7 (Bitplane, Switzerland), respectively.

### RNA isolation and quantitative real-time PCR

The tissue or cell samples were dissolved and immersed in Trizol reagent (Ambion, USA) for total RNA extraction. The cDNA of RNA samples was obtained by following the reverse transcription procedure of the PrimeScript RT reagent kit with gDNA Eraser (Takara, China). Quantitative real-time PCR was carried out using TB Green Premix Ex Taq II (Takara, China) on a QuantStudio 5 system (Applied Biosystems, USA). The expression level of target genes was calculated by the 2^−ΔΔCT^ method normalized to the reference gene glyceraldehyde-3-phosphate dehydrogenase (*Gapdh*) The primers of target genes are listed in Table S1 (see online supplementary material)

### Western blotting

At the scheduled time point, protein samples were first harvested after lysing cells with Sodium dodecyl sulfate (SDS) lysis buffer containing protease inhibitors (Roche). Subsequently, samples were separated by 10% SDS-polyacrylamide gel electrophoresis and transferred to a methanol-activated polyvinylidene difluoride membrane (GE Healthcare, USA). Then, the membrane was incubated with TBST buffer containing 5% bovine serum albumin and probed with anti-CTHRC1 antibody (1:600, ab85739, Abcam, USA) overnight at 4°C. The membranes were incubated with anti-β-actin (1:5000, 66 009–1-Ig, Proteintech, USA) or anti-GAPDH (1:5000, 60 004–1-Ig, Proteintech, USA) as the loading control. Finally, the protein expression level was visualized with an enhanced chemiluminescence (ECL) detection system (Applygen, China) in a UVITEC Alliance MINI HD9 instrument (UVITEC, UK).

### RNA sequencing

To obtain dermal tissue, freshly harvested mouse skin was digested with 2.5 mg/ml dispase II (Gibco, USA). Total RNA was extracted from the specimen using an Animal Tissue RNA Purification Kit (Norgen, Canada). Genes with fold change >2.0, false rate < 0.05 and mean log intensity >2.0 were considered significant. Gene ontology (GO) enrichment analysis of differentially expressed genes was carried out with the Metascape online tool (https://metascape.org/gp/index.html#/main/step1).

### Cell isolation and culture

Primary DMECs were isolated from the 1-day-old mouse skin using a modified protocol [25]. Briefly, freshly harvested mouse skin was digested with 2.5 mg/ml dispase II at 4°C for 12 h. The dermal tissue was separated from the epidermal layer by mechanical dissociation, minced, and digested with 4 mg/ml type I collagenase at 37°C for 2 h. Following filtration and centrifugation, the cell pellet was resuspended in EGM-2 (MV Microvascular Endothelial Cell Growth Medium-2 BulletKit; CC-3162, Lonza, Switzerland) containing 1 μg/ml puromycin (Solarbio, China) and clutured for 50 h at 37°C and 5% CO_2_. Afterwards, the medium was replaced with EGM-2 without puromycin and continuously cultured for 1 week. The cobblestone-shaped cells appeared as early as days 3. The cells were treated at passage 3 and the medium was changed every 3 days. Primary DMECs were confirmed by positive CD31 (ab28364, Abcam, 1:50), factor VII (ab203590, Abcam, 1:100) and *Bandeiraea simplicifolia* lectin (BSL) (L9381, Sigma-Aldrich, 10 μg/ml) staining and their ability to uptake DiI-labeled acetylated low-density lipoprotein (DiI-Ac-LDL; Solarbio, 30 μg/ml). The potential of DMECs to form functional microvessels was also confirmed by positive zonula occludens-1 (ZO-1) (61–7300, Invitrogen, 1:100) staining.

### Flow cytometry

The cell purity of DMECs was identified by flow cytometry. The cells were harvested, washed twice with PBS and incubated with fluorescein isothiocyanate (FITC)-conjugated Factor VIII monoclonal antibody (BD Bioscience, USA) for 1 h at 4°C in the dark. Then the cells were detected using a flow cytometer (BD Bioscience, USA).

### Tube-formation assay

Tube-formation assay was carried out to assess the proangiogenic ability of CTHRC1. DMECs were first harvested and resuspended in an EGM-2 medium containing 300 ng/ml mouse CTHRC1 recombinant protein (rmCTHRC1; AdipoGen, USA). Then the cells were seeded on the Matrigel at a density of 2.5 × 10^5^ cells/ml. After 10 h of incubation, the tube-like network of DMECs was captured with a phase-contrast microscope (Leica, Germany). The tube length and node number were measured with ImageJ software version 1.53 k (NIH, USA).

### Rescue assay and laser Doppler imaging

*Cthrc1* ^−/−^ mice (both male and female) were 6–8 weeks of age and were anesthetized with 4% (w/v) chloral hydrate. PBS (30 μL) or rmCTHRC1 (600 ng/ml) was subcutaneously injected into the hind paws at 0 and 3 days. Blood flow of the palm pads was measured using a laser Doppler perfusion imaging system (Perimed, Sweden) post 0, 3 and 7 days. Three measurements were averaged after scanning the same regions of interest (ROIs). The perfusion signal was displayed in a color scale bar ranging from dark blue (low perfusion) to red (high perfusion). Results were normalized to the WT or the PBS group at 0 days.

### Histological and immunofluorescent staining

At the scheduled time point, the hind feet were isolated from the anesthetized mice, fixed in 4% paraformaldehyde, decalcified and embedded in paraffin following standard procedures. Hematoxylin and eosin (H&E) staining were carried out to visualize the anatomical structure of SGs and their surrounding microvessels. For immunofluorescent imaging, the slide samples were first rinsed with PBS and then treated with 10% normal goat serum (Solarbio, China) for 1 h to block nonspecific epitopes. After that, the samples were incubated with primary antibodies at 4°C overnight for anti-keratin 14 (K14, ab7800, Abcam, 1:200), anti-keratin 18 (K18, ab668, Abcam, 1:200), anti-keratin 19 (K19, ab52625, Abcam, 1:200), anti-ATPase Na^+^/K^+^ transporting subunit alpha 1 (ATP1a1, ab7671, Abcam, 1:200), anti-VE-cadherin (sc-9989, Santa Cruz, 1:100), anti-CD31 (ab28364, Abcam, 1:50), anti-ZO-1 (1:100) and anti-laminin (ab11575, Abcam, 1:100). Subsequently, the secondary antibodies were incubated with samples at room temperature for 2 h in the dark as follows: CoraLite488 conjugated goat anti-mouse IgG (SA00013–1, Proteintech, USA, 1:200), CoraLite488 conjugated goat anti-rabbit IgG (SA00013–2, Proteintech, USA, 1:200), CoraLite594 conjugated goat anti-mouse IgG (SA00013–3, Proteintech, USA, 1:200) and CoraLite594 conjugated goat anti-rabbit IgG (SA00013–4, Proteintech, USA, 1:200). Cell nuclei were labeled with 4′,6-diamidino-2-phenylindole (DAPI; Sigma-Aldrich, USA). Fluorescent images were acquired with a TCS SP8 confocal microscope (Leica, Germany).

### Statistical analysis

At least three replicates were conducted in all experiments. Sample sizes are given in the figure legends. Results are presented as mean ± standard deviation (SD). Statistical analysis was performed using GraphPad Prism 7.0 (GraphPad, USA). Two-group comparison was performed by unpaired two-tailed *t*-test and multiple-group comparison was performed by one-way or two-way analysis of variance (ANOVA) with Tukey’s multiple comparisons test. Group significance level was noted as ^*^p < 0.05, ^*^^*^p < 0.01, ^*^^*^^*^p < 0.001, ^*^^*^^*^^*^p < 0.0001. 

**Figure 1.**
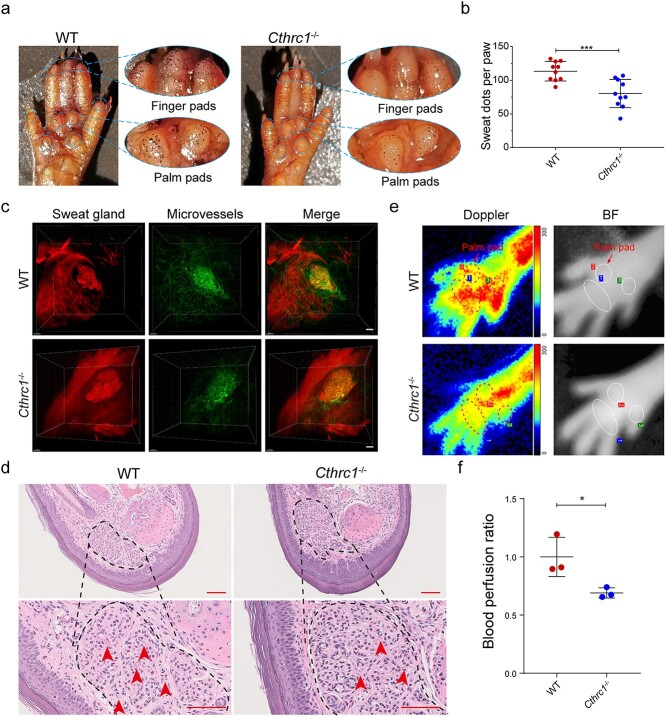
The role of CTHRC1 in SG function. (**a**) Sweat test of WT and *Cthrc1^−/−^* mice. Dashed boxes indicate sweaty areas of mice paw pads. (**b**) Quantification of sweat dots on the paw pads of WT and *Cthrc1^−/−^* mice. Each dot in the statistical graph represents the number of dark dots on one hind paw from an individual mouse (*n* = 10). (**c**) Light-sheet microscopy images showing the SGs and their surrounding microvascular networks of WT and *Cthrc1^−/−^* mice. 3D reconstruction of light-sheet microscopy images was preformed using Imaris software (Scale bar: 100 μm). (**d**) Representative H&E images of SGs and their adjacent microvessels. Arrowheads highlight the location of microvessels (Scale bar: 100 μm, top row; Scale bar: 25 μm, bottom row). (**e**) Representative images of laser Doppler blood flow perfusion and corresponding BF of WT and *Cthrc1^−/−^* mice. Dashed boxes are at the same location as the ROIs in BF and indicate the palm pads of the mice left hind paws. (**f**) Analysis of the blood flow of the ROIs of WT and *Cthrc1^−/−^* mice (*n* = 3). Results were normalized to the WT mice. Results are presented as the mean ± SD, ^*^p < 0.05, ^*^^*^^*^p < 0.001. *CTHRC1* Collagen triple helix repeat containing-1 protein, *Cthrc1* collagen triple helix repeat containing-1 gene, *SG* sweat gland, *WT* wild type, *H&E* hematoxylin and eosin, *BF* bright field, *ROIs* regions of interest, *SD* standard deviation

## Results

### Role of CTHRC1 in SG function

To identify the role of CTHRC1 in SG function, we first analyzed the effects of *Cthrc1* gene deletion on SG function by sweat test in WT and *Cthrc1*^−/−^ mice. Since SGs are restricted to the paw pads in mice [1], we tested sweat secretion in the hind paws of mice, which provide the major interdigital pads. After 10 min of acetylcholine injection, dark dots on the finger and palm pads corresponded to the active sweat pores (Figure 1a). Compared with the WT mice, the number of dark dots on the pad surface of *Cthrc1*^−/−^ mice was siginficantly reduced. This suggested that *Cthrc1*^−/−^ mice have a weaker sweating function (Figure 1b). Considering the dual role of CTHRC1 in promoting SG development and tissue vascularity, we further compared the differences in the anatomical structure of SGs and microvessels between WT and *Cthrc1*^−/−^ mice. 3D reconstruction of light-sheet microscopy images displayed a complicated, well-organized and high-density capillary network surrounding SGs in WT mice (Figure 1c and Video 1, see online supplementary material). 3D images also revealed a disordered and low-density capillary network near SGs in *Cthrc1*^−/−^ mice, although the loops of sweat tubules showed no significant difference compared to the WT mice (Figure 1c and Video 2, see online supplementary material). At the same time, H&E staining also showed that the number of microvessels in the sweat tubule gaps after *Cthrc1* knockout was less than that in WT mice (Figure 1d). Given this, we next evaluted the function of vascular networks around SGs in *Cthrc1*^−/−^ mice. The blood flow of major interdigital pads (ROI 1, 2 and 3) was scanned with a deep penetrating laser Doppler probe. As shown in Figure 1e, f**,** the blood flow perfusion of *Cthrc1*^−/−^ mice was significantly lower than that in the WT mice. These results indicated that deletion of *Cthrc1* attenuated SG function, which may be related to a reduced vascular network.

### Role of CTHRC1 in the development of SGs and their adjacent microvasculature

**Figure 2.**
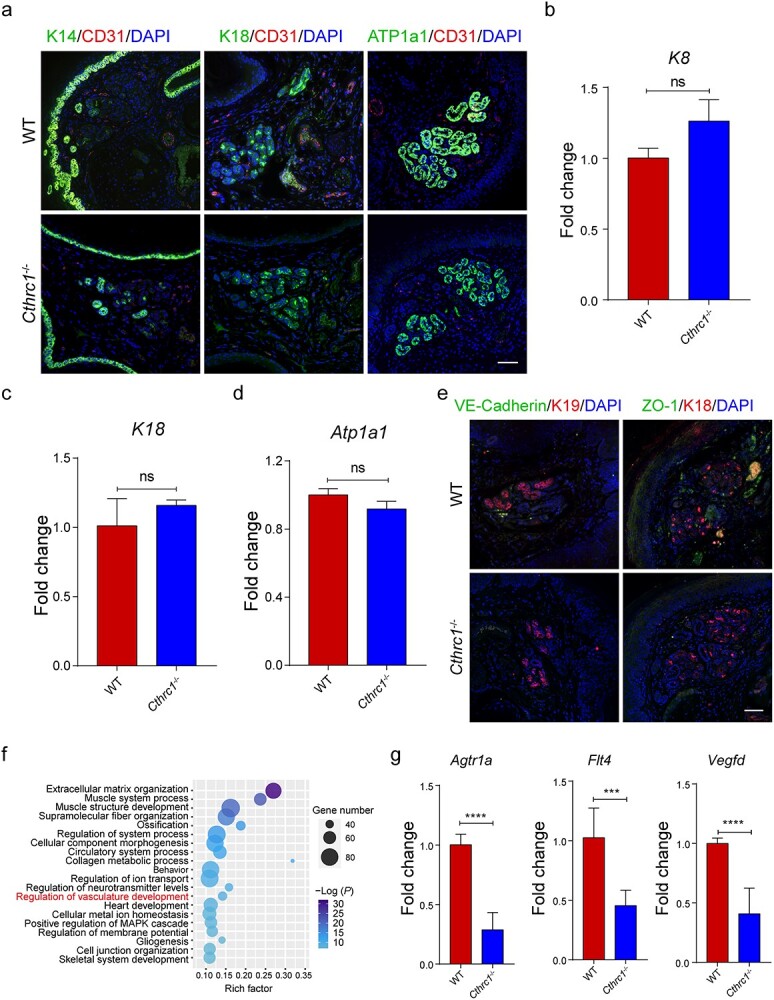
The role of CTHRC1 in the development of SGs and their adjacent vasculature. (**a**) Comparison of SG markers K14 and K18 and secretion-related marker ATP1a1 between WT and *Cthrc1^−/−^* mice. K14, K18 and ATP1a1, green; CD31, red; DAPI, blue (Scale bar: 50 μm). (**b**–**d**) The relative expression values of *K8*, *K18* and *Atp1a1* mRNA of SGs of WT and *Cthrc1^−/−^* mice (*n* = 3). (**e**) Comparison of vasculature formation around SGs by staining for endothelial adherens (VE-cadherin) and tight junctions’ marker (ZO-1). VE-Cadherin and ZO-1, green; K18 and K19, red; DAPI, blue (Scale bar: 50 μm). (**f**) Scatter plot showing the down-regulated genes in the *Cthrc1^−/−^* mice compared to WT mice by GO enrichment analysis (top 20 GO terms). The rich factor represents the ratio of the down-regulated genes to all genes enriched in the corresponding GO term. Dot size represents the number of down-regulated genes enriched in a specific GO term. Dot color represents the *P* value obtained by GO analysis. *P* < 0.05 was used to indicate significant enrichment. (**g**) The relative expression values of *Agtr1a, Flt4* and *Vegfd* mRNA in dermal tissue of WT and *Cthrc1^−/−^* mice (*n* = 3). Results are presented as the mean ± SD, ^*^^*^^*^p < 0.001, ^*^^*^^*^^*^p < 0.0001; ns, not significant. *CTHRC1* Collagen triple helix repeat containing-1 protein, *Cthrc1* collagen triple helix repeat containing-1 gene, *SG* sweat gland, *K* cytokeratin, *ATP1a1* ATPase Na^+^/K^+^ transporting subunit alpha 1, *CD31* platelet and endothelial cell adhesion molecule 1, *WT* wild type, *mRNA* messenger RNA, *VE-cadherin* vascular endothelial cadherin, *ZO-1* zonula occludens-1, *DAPI* 4′,6-diamidino-2-phenylindole, *GO* gene ontology, *MAPK* mitogen-activated protein kinase, *Agtr1a*, angiotensin II receptor type 1a, *Flt4* fms related receptor tyrosine kinase 4, *Vegfd* vascular endothelial growth factor D, *SD* standard deviation

We next investigated the effect of *Cthrc1* deletion on the gene and protein expression levels of SGs and their adjacent microvasculature. Representative fluorescent images showed no significant difference in the expression of SG markers (K14 and K18) and secretion marker (ATP1a1) between WT and *Cthrc1*^−/−^ mice (Figure 2a). Then, we manually isolated SGs from the palm pads after mild digestion with collagenase type I and detected the expression level of SG marker genes (*K8* and *K18*) and functional genes (*Atp1a1*, *Fxyd2*, *Aqp5* and *Atp1b1*). Statistical results also showed that the expression level of these genes in *Cthrc1*^−/−^ mice were comparable with the WT mice (Figure 2b-d and Figure S1, see online supplementary material). Next, we assessed the specific marker (CD31) and functional markers (VE-cadherin and ZO-1) expression levels of microvessels surrounding SGs. Compared with the WT mice, the number of CD31^+^, VE-cadherin^+^ and ZO-1^+^ microvessels in the SG gaps was significantly reduced in *Cthrc1*^−/−^ mice (Figure 2a, e). To further elucidate the underlying mechanism of this phenotype, we performed RNA sequencing analysis using the dermal tissue of WT and *Cthrc1*^−/−^ mice. GO enrichment analysis of differentially expressed genes was performed based on the sequencing results (Figure 2f). The scatter plot displayed that most down-regulated genes in *Cthrc1*^−/−^ mice were related to ECM organization, collagen metabolism and regulation of vasculature development. Focusing on the role of *Cthrc1* in vascular development, we identified three genes involved in the GO term of regulation of vasculature development. Quantitative real-time PCR analysis showed that the gene expression level of *Agtr1a*, *Flt4* and *Vegfd* in palm pads dermis of *Cthrc1*^−/−^ mice was significantly down-regulated compared with WT mice (Figure 2g). In short, our data suggested that *Cthrc1* gene deletion did not affect the expression of functional genes in SGs, but hindered the formation and maturation of the vascular network around the SGs.

### Identification of WT and *Cthrc1*^*−/−*^ mice-derived DMECs

**Figure 3.**
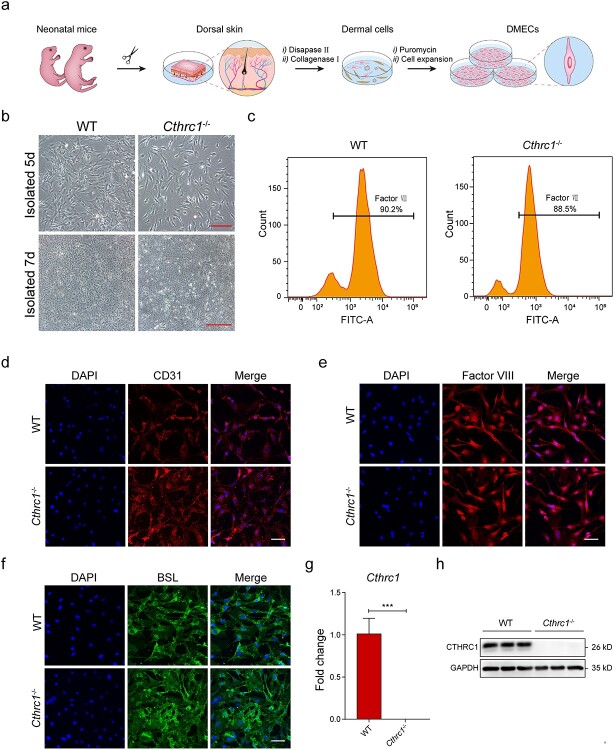
Identification of WT and *Cthrc1^−/−^* mice-derived DMECs. (**a**) Schematic overview of the procedure carried out to isolate and purify DMECs. (**b**) Morphological properties of DMECs at days 5 and 7 (Scale bar: 100 μm, top row; Scale bar: 500 μm, bottom row). (**c**) Factor VII flow cytometry analysis of the positive rate of DMECs. DMECs displaying positive staining for (**d**) CD31 (Scale bar: 50 μm), (**e**) factor VII (Scale bar: 50 μm) and (**f**) BSL (Scale bar: 50 μm). (**g**) The relative expression values of *Cthrc1* mRNA in DMECs (*n* = 3). (**h**) Western blot analysis showing the CTHRC1 expression level of DMECs (*n* = 3). Results are presented as the mean ± SD, ^*^^*^^*^p < 0.001. *Cthrc1* Collagen triple helix repeat containing-1 gene, *WT* wild type, *DMECs* dermal microvascular endothelial cells, *CD31* platelet and endothelial cell adhesion molecule 1, *BSL Bandeiraea simplicifolia* lectin, *mRNA* messenger RNA, *CTHRC1* collagen triple helix repeat containing-1 protein, *SD* standard deviation

To systematically study the regulatory role of CTHRC1 in dermal microvascular development at the cellular level, we first isolated the DMECs from dermal tissues of WT and *Cthrc1^−/−^* mice using a modified protocol (Figure 3a) [25]. At the initial isolation stage, both DMECs exhibited a typical ‘cobblestone’ appearance. On days 5–7, the round DMECs gradually became more extensive, pseudopodia stretched, turned into the spindle and polygonal shape and contributed to tightly fused cell colonies (Figure 3b). Factor VII flow cytometry analysis showed that the cell purity of both DMECs was >88% (Figure 3c). Endothelial markers of DMECs were also detected by immunofluorescent staining and lectin-binding experiments. Representative fluorescent images showed no significant difference in the expression of CD31 and Factor VII between WT and *Cthrc1*^−/−^ DMECs (Figure 3d, e). Previous research has shown that rodent (but not human) microvascular endothelial cells showed a high level of binding intensity for BSL [26]. After incubating the isolated DMECs with FITC-conjugated BSL for 1 h, we found apparent positive staining on the surface of both cells (Figure 3f). Next, we examined the expression level of the *Cthrc1* gene in WT and *Cthrc1^−/−^* DMECs. Quantitative real-time PCR analysis showed that the *Cthrc1* gene in *Cthrc1*^−/−^ DMECs was not expressed compared with WT DMECs (Figure 3g). Western blot analysis also confirmed that the CTHRC1 protein was not expressed in *Cthrc1*^−/−^ DMECs (Figure 3h).

**Figure 4.**
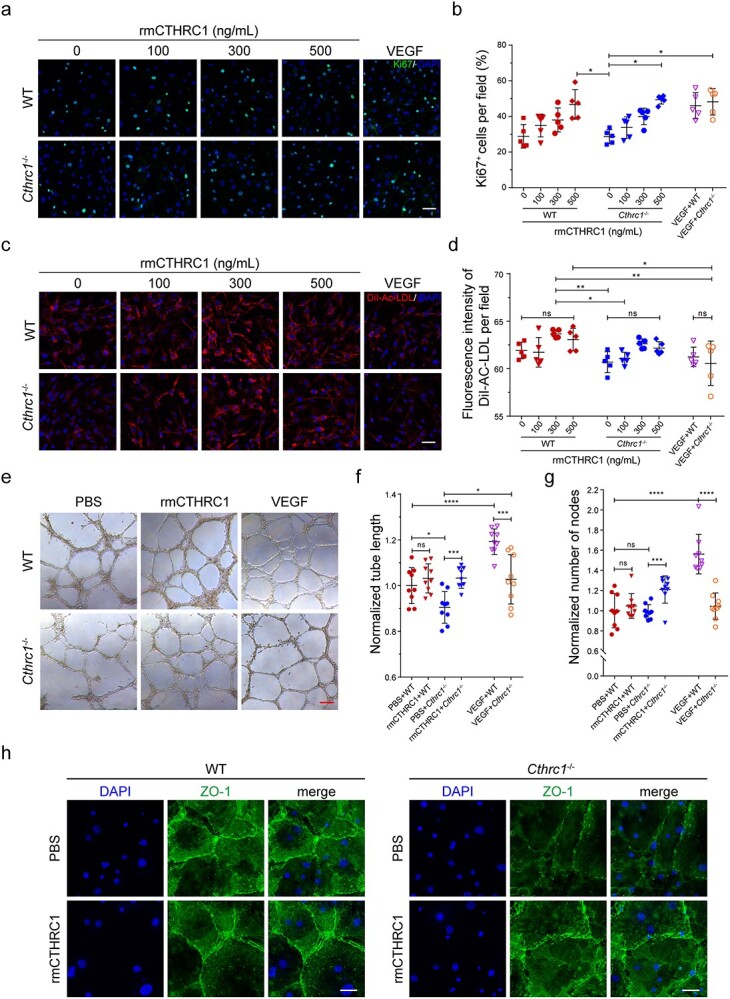
Regulation of CTHRC1 of the angiogenic ability of DMECs derived from WT and *Cthrc1^−/−^* mice. (**a**) Immunofluorescence analysis of Ki67 expression of DEMCs after treatment with different concentrations of rmCTHRC1. The addition of VEGF (50 ng/ml) was used as a positive control. Ki67, green; DAPI, blue (Scale bar: 50 μm). (**b**) Quantification of the rate of Ki67^+^ DMECs in randomly acquired confocal images (*n* = 5). (**c**) Florescent imaging displaying DiI-Ac-LDL uptake by DMECs in response to different concentrations of rmCTHRC1 stimuli. The addition of VEGF (50 ng/ml) was used as a positive control. DiI-AC-LDL, red; DAPI, blue (Scale bar: 50 μm). (**d**) Quantify the geometric mean fluorescence intensity of DiI-AC-LDL uptake by DMECs in randomly acquired confocal images (*n* = 5). (**e**) Phase-contrast images of the capillary-like structures of DMECs treated with rmCTHRC1 (300 ng/ml) after 10 h. The addition of VEGF (50 ng/ml) and PBS was used as a positive and negative controls, respectively (Scale bar: 200 μm). (**f**, **g**) Quantification of tube formation degree of DMECs using tube length and nodes number (n = 9). (**h**) Immunofluorescence analysis of intercellular junction formation of DMECs treated with rmCTHRC1 (300 ng/ml) after 3 days (Scale bar: 100 μm). Results are presented as the mean ± SD, ^*^p < 0.05, ^*^^*^p < 0.01, ^*^^*^^*^p < 0.001, ^*^^*^^*^^*^p < 0.0001; ns, not significant. *CTHRC1* Collagen triple helix repeat containing-1 protein, *Cthrc1* collagen triple helix repeat containing-1 gene, *WT* wild type, *DMECs* dermal microvascular endothelial cells, *Ki67* antigen identified by monoclonal antibody Ki 67, *rmCTHRC1* mouse CTHRC1 recombinant protein, *VEGF* vascular endothelial growth factor, *DAPI* 4′,6-diamidino-2-phenylindole, *DiI-Ac-LDL* DiI-labeled acetylated low-density lipoprotein, *PBS* phosphate buffered saline, *SD* standard deviation

**Figure 5.**
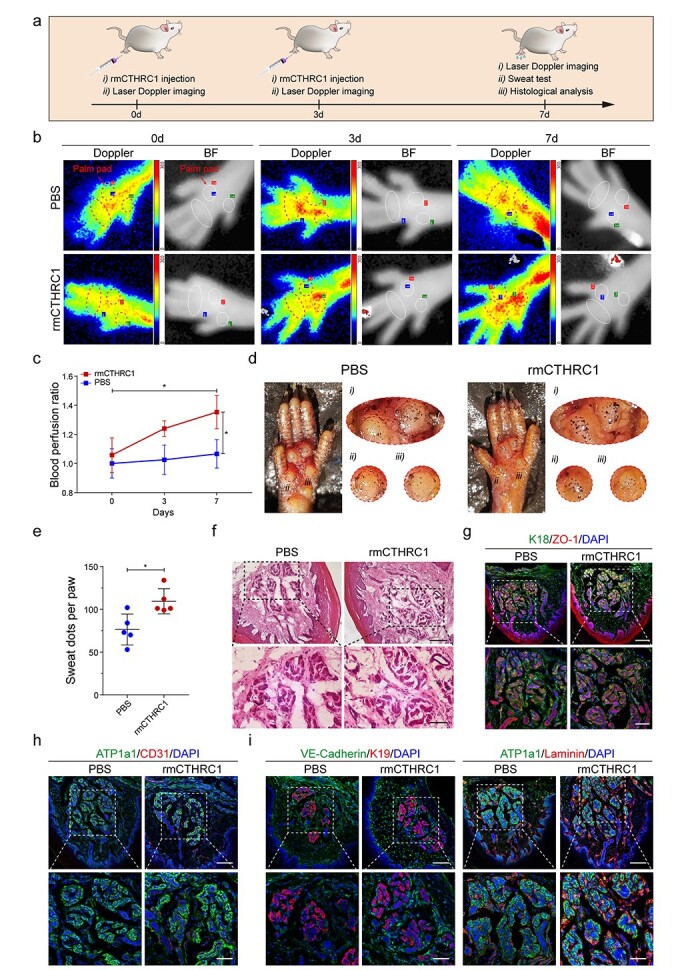
rmCTHRC1 promotes the recovery of SG function by inducing adjacent microvascular network remodeling. (**a**) Workflow for rmCTHRC1 injection and evaluating the blood flow perfusion and SG function recovery of *Cthrc1^−/−^* mice. (**b**) Representative images of laser Doppler blood flow and corresponding BF of *Cthrc1^−/−^* mice treated with rmCTHRC1 (600 ng/ml) for 0, 3 and 7 days. The injection of PBS was used as a negative control. Dashed boxes are at the same location as the ROIs in BF and indicate the palm pads of the mice left hind paws. (**c**) Analysis of the blood flow of the ROIs (*n* = 3). Results were normalized to the PBS group at 0 days. (**d**) Sweat test of *Cthrc1^−/−^* mice treated with rmCTHRC1 for 7 days. The PBS group was used as a control. Dashed boxes indicate sweaty areas of mouse paw pads (at the same location as the ROIs in BF). (**e**) Quantification of sweat dots on the paw pads of *Cthrc1^−/−^* mice. Each dot represents the number of dark dots on one hind paw from an individual mouse (*n* = 5). (**f**, **g**) Representative H&E and immunofluorescence images of SGs of *Cthrc1^−/−^* mice treated with rmCTHRC1 or PBS for 7 days (Scale bar: 100 μm, top rows; Scale bar: 50 μm, bottom rows). (**h**, **i**) Immunofluorescence analysis of the number (CD31), endothelium junction (VE-cadherin), and basement membrane (laminin) of SG adjacent microvessels. K18, ATP1a1, VE-cadherin, green; ZO-1, CD31, K19, Laminin, red; DAPI, blue (Scale bar: 100 μm, top rows; Scale bar: 50 μm, bottom rows). Results are presented as the mean ± SD, ^*^p < 0.05. *rmCTHRC1* Mouse CTHRC1 recombinant protein, *SG* sweat gland, *Cthrc1* collagen triple helix repeat containing-1 gene, *BF* bright field, *PBS* phosphate-buffered saline, *ROIs* regions of interest, *H&E* hematoxylin and eosin, *CD31* platelet and endothelial cell adhesion molecule 1, *VE-cadherin* vascular endothelial cadherin, *K* cytokeratin, *ATP1a1* ATPase Na^+^/K^+^ transporting subunit alpha 1, *ZO-1* zonula occludens-1, *DAPI* 4′,6-diamidino-2-phenylindole, *SD* standard deviation

### Regulation by CTHRC1 of the angiogenic ability of DMECs

After successful isolation and culture of dermis-specific endothelial cells, we further investigated the effect of CTHRC1 on the angiogenic activity of DMECs *in vitro*. Considering the critical role of EC proliferation in angiogenesis, we first evaluated the effect of CTHRC1 on DMEC proliferation. After incubating the DMECs with 0, 100, 300 and 500 ng/ml rmCTHRC1 for 3 days, the number of proliferating Ki67^+^ cells of *Cthrc1^−/−^* and WT DMECs in the high-concentration group (500 ng/ml) was significantly higher than that in the low-concentration group (0 ng/ml; Figure 4a, b). However, the proliferation rate (Ki67^+^ cells per field) of both DMECs showed no difference. These results imply that the CTHRC1 has a remarkable promoting effect on DMEC proliferation. After that, we further assessed the effect of CTHRC1 on DMEC function. Ac-LDL uptake, tube formation and intercellular junction establishment are crucial function indexes for ECs [27, 28]. Representative fluorescent images displayed that DMEC endocytosis of Ac-LDL is CTHRC1 concentration-dependent (Figure 4c). Quantitative analysis showed that large amounts of Ac-LDL were taken up by both DMECs incubated with 300 ng/mL rmCTHRC1, whereas only a tiny amount was observed in DMECs incubated without rmCTHRC1 (Figure 4d). Furthermore, the statistical results also indicated that the WT DMECs showed a greater absorbed quantity of Ac-LDL than *Cthrc1^−/−^* DMECs, even though there was no rmCTHRC1 administration. Subsequently, we evaluated the effect of CTHRC1 on DMEC tube formation based on phase-contrast images (Figure 4e). Compared with the WT DMECs, the tube-forming ability of *Cthrc1^−/-^*DMECs was considerably decreased after *Cthrc1* deletion (PBS group), while this angiogenesis ability was significantly restored after the application of rmCTHRC1 (Figure 4f, g). Finally, we investigated the effect of CTHRC1 on the intercellular junction establishment of DMECs. The ECs of functional microvascular networks are positive staining for some vascular markers, i.e. ZO-1 [27]. To observe the potential of DMECs to establish intercellular junctions in different conditions, we incubated DMECs with rmCTHRC1 or PBS for 3 days. In the PBS group, there was only a sporadic expression of ZO-1 in the boundary of *Cthrc1^−/−^* DMECs relative to WT DMECs. Conversely, the cell–cell junctions of *Cthrc1^−/−^* DMECs were reestablished in some regions after rmCTHRC1 addition (Figure 4h).

### rmCTHRC1 promotes the recovery of SG function by inducing adjacent microvascular network reconstruction

To further verify the repair capacity of CTHRC1 *in vivo*, we replenished rmCTHRC1 into the paws of *Cthrc1*^−/−^ mice. The workflow for rmCTHRC1 injection and evaluating the blood flow perfusion and SG function recovery is summarized in Figure 5a. We first assessed the function of the vascular network around SGs in palm pads on days 0, 3 and 7 (Figure 5b). Compared with the PBS group, the signals of blood flow perfusion in major interdigital pads, especially ROI 1 and 3, were significantly enhanced after rmCTHRC1 administration for 7 days (Figure 5b, c). Subsequently, we further evaluated the SG function repair effect using the iodine/starch-based sweat test on day 7. After 10 min of acetylcholine injection, a large number of dark dots appeared on the surface of the palm pads (Figure 5d). Quantitative analysis showed that the number of dark dots on the pad surface of rmCTHRC1-treated mice dramatically increased relative to that before treatment. In contrast, there were still only a few dark dots on the pad surface of the PBS-treated group (Figure 5e and Figure 1b). Finally, we also confirmed the repair effect of SG function by histological and immunofluorescent staining. After 7 days of treatment, H&E staining and colocalization analysis of K18 and ZO-1 showed that the morphology of sweat tubules in rmCTHRC1-treated mice had no changes relative to the control group (Figure 5f, g). On the contrary, the number of microvessels in the sweat tubule gaps of treated mice were clearly increased (Figure 5h). In addition, the considerable increase of VE-cadherin^+^ and laminin^+^ microvessels also supported the hypothesis that a more mature and functional microvascular network was established surrounding the SGs of rmCTHRC1-treated mice (Figure 5i).

## Discussion

CTHRC1 was first identified in balloon-injured rat arteries and is mainly involved in the vascular remodeling process and angiogenesis by regulating cell migration and collagen synthesis [13, 16]. Recently, an increasing number of studies have indicated a close correlation between CTHRC1 and wound healing [19], skin appendage specification [21], patterning [20] and regeneration [9]. Our previous findings verified that CTHRC1 was a crucial biochemical regulator. It could direct the conversion of mesenchymal stem cells, mammary progenitor cells and epidermal progenitors into functional SGs in a 3D bioprinted matrix [12, 21, 29, 30]. However, although CTHRC1 is a critical protein in the SG microenvironment, its biological role in the development, morphogenesis and function execution of SGs and their neighboring tissues *in vivo* is still unknown. Given this, we initially determined the role of CTHRC1 in SG function using sweat test, laser Doppler imaging and 3D reconstruction of SGs and their adjacent microvascular network in WT and *Cthrc1*^−/−^ mice. Our findings showed that CTHRC1 played a crucial role in sweat secretion in mice. *Cthrc1* deletion had a few effects on SG morphogenesis but resulted in the disorder and reduction of the microvascular network around SGs. These results highlight the promoting effect of CTHRC1 on the construction of vascular networks around SGs.

Considering the prominent role of CTHRC1 in blood vessel repair and promoting tissue vascularity [15, 31], we further investigated the *Cthrc1* deletion effect on the expression of vascular development genes and functional proteins using transcriptome and colocalization analysis. Although knockout of *Cthrc1* had few effects on SGs, it dramatically reduced the expression of vascular development genes and functional proteins in the dermal tissues of *Cthrc1*^−/−^ mice. Furthermore, CTHRC1 is known to participate in the regulation of collagen synthesis and even cardiac and pulmonary fibrosis [14, 32, 33]. Therefore, GO analysis of dermal transcriptomic data showed that the majority of down-regulated genes in *Cthrc1*^−/−^ mice were also involved in ECM organization and collagen metabolism. In addition, a large amount of collagen (i.e. basal lamina) exists around the endothelium of mature blood vessels to maintain the stability of the vascular structure [34]. *Cthrc1* deletion may hinder basal lamina formation and lead to the structural disorder and reduced density of the microvascular network around SGs in *Cthrc1*^−/−^ mice.

DMECs, considered tissue-specific and more responsive to angiogenic factors, have gradually become a vital cell model in skin research [25, 35]. Due to low proliferation rates, primary DMECs are challenging to isolate and are often contaminated and displaced by fibroblasts or other mesenchymal cells [35]. To systematically study the regulatory role of CTHRC1 in dermal microvascular development, we isolated the DMECs from dermal tissues of neonatal mice using a sample and repeatability protocol [25, 36]. The cell purity of DMECs was >88%. After successful isolation and culture, we further investigated the effect of CTHRC1 on the angiogenic activity of these dermis-specific endothelial cells *in vitro*, i.e. cell proliferation, DiI-Ac-LDL uptake, tube formation and intercellular junction establishment. We identified CTHRC1 as a potential key regulator for the angiogenic ability of DMECs. After *Cthrc1* deletion, the vessel-forming ability of DMECs was decreased considerably, while this ability was significantly restored after the application of CTHRC1.

Sweating is an essential physiological process in thermoregulation for our bodies [1]. However, the skin and its appendages (i.e. SGs) are often partially or entirely lost in severe burns or trauma [9]. SGs have low regenerative potential in response to deep dermal injury and their regeneration or functional restoration still faces many challenges [11, 12, 37]. Stem-cell-based strategies have advanced SG regeneration, yet functional regeneration has not been achieved [29, 30, 38, 39]. Blood vessels are a neighboring vital tissue for SGs and are considered to support the reabsorption of sweat components [7, 8, 23]. Understanding the relationship between SGs and their specific vasculature largely facilitates SG functional restoration [6, 11, 40]. Given this, to further verify the repair capacity of CTHRC1 *in vivo*, we replenished the CTHRC1 into the paws of *Cthrc1*^−/−^ mice at the scheduled time. After CTHRC1 administration for 7 days, although the morphology of sweat tubules in CTHRC1-treated mice showed no changes relative to the control group, the SG function was significantly enhanced. Furthermore, the blood flow perfusion in major interdigital pads was meaningfully pronounced, and a denser, more mature and functional microvascular network was established surrounding the SGs. In short, all these results imply that CTHRC1 administration may promote the recovery of SG function by inducing adjacent microvascular network reconstruction.

## Conclusions

*Cthrc1* gene plays a crucial role in sweat secretion in mice. Macroscopically, *Cthrc1* deletion had few effects on SG morphogenesis but resulted in the disorder and reduction of the microvascular network around SGs. Microscopically, knockout of *Cthrc1* had no effect on the expression of SG marker genes and proteins but reduced the expression of vascular development genes and functional proteins in the dermal tissues. CTHRC1 promotes the recovery of SG function by inducing adjacent microvascular network reconstruction.

## Abbreviations

*Agtr1a*: angiotensin II receptor type 1a; *ATP1a1*: ATPase Na^+^/K^+^ transporting subunit alpha 1; *BF*: bright field; *BSL*: *Bandeiraea simplicifolia* lectin; *CD31*: platelet and endothelial cell adhesion molecule 1; *Cthrc1*: collagen triple helix repeat containing-1 gene; *CTHRC1*: collagen triple helix repeat containing-1 protein; *DAPI*: 4′,6-diamidino-2-phenylindole; *DiI-Ac-LDL*: DiI-labeled acetylated low-density lipoprotein; *DMECs*: dermal microvascular endothelial cells; ECM: extracellular matrix; EGM-2: endothelial cell growth medium-2; *Flt4*: fms related receptor tyrosine kinase 4; *GO* :gene ontology; HF: hair follicle; *H&E*: hematoxylin and eosin; *K*: cytokeratin; *Ki67*: antigen identified by monoclonal antibody Ki 67; *MAPK*: mitogen-activated protein kinase; *mRNA*: messenger RNA; *PBS*: phosphate-buffered saline; *rmCTHRC1*: mouse CTHRC1 recombinant protein; *ROI* : regions of interest; *SD*: standard deviation; *SG*: sweat gland; *VE-cadherin*: vascular endothelial cadherin; *VEGF*: vascular endothelial growth factor; *WT*: wild type; *Vegfd*: vascular endothelial growth factor D; *ZO-1*: zonula occludens-1.

## Data availability

The data underlying this article will be shared on reasonable request to the corresponding author.

## Authors’ contributions

XY and SH designed the experiments and wrote the manuscript. XY, XD, ZL, FZ and LL performed the experiments. XY, E, WS, SZ, and MZ analyzed the data. XY, XD, BY, YK, and SH performed the statistical analysis and revised the manuscript. YW, CZ, and XF contributed reagents, materials and analysis tools. XF and SH provided equipment and research funds and approved the final submission. All authors read and approved the final manuscript.

## Supplementary Material

Supplementary_data_R2_tkac035

Supplementary_Movie_1_tkac035

Supplementary_Movie_2_tkac035
