# Supplementary material for: Collagen triple helix repeat containing-1 promotes functional recovery of sweat glands by inducing adjacent microvascular network reconstruction in vivo
Source: Burns Trauma. 2022 Aug 2;10:tkac035. doi: 10.1093/burnst/tkac035 (PMC9346565; doi:10.1093/burnst/tkac035)
Supplement: Supplementary_data_R2_tkac035 [file supplementary_data_r2_tkac035.docx]

Supplementary data

**CTHRC1 promotes functional recovery of sweat gland by inducing adjacent microvascular network remodeling**

**Table of Contents**

SI-1. Descriptions of Supplementary tables

SI-2. Captions of Supplementary Movies 1-2

SI-3. The expression levels of SG function genes in mice [Figure S1]

**SI-1. Descriptions of Supplementary tables**

**Table S1. Primers of target genes used in this study.**

| **Primer** | **Sequence (5’ to 3’)** |
| --- | --- |
| Krt8-F | AGAAGGATGTGGACGAAGCA |
| Krt8-R | ACGAACTTCAGCGATGATGC |
| Krt18-F | ACTCCGCAAGGTGGTAGATGA |
| Krt18-R | TCCACTTCCACAGTCAATCCA |
| Atp1a1-F | GGACGACCATAAACTCAGCCT |
| Atp1a1-R | GCAGGTGTTAATCCTCGGCTC |
| Fxyd2-F | AGAGAATCCCTTCGAGTACGAC |
| Fxyd2-R | GCGGAACCTTTTGCTGAGAATG |
| Aqp5-F | TCTTGTGGGGATCTACTTCACC |
| Aqp5-R | TGAGAGGGGCTGAACCGAT |
| Atp1b1-F | GCTGCTAACCATCAGTGAACT |
| Atp1b1-R | GGGGTCATTAGGACGGAAGGA |
| Cthrc1-F | GCTGTCAGCGCTGGTATTTT |
| Cthrc1-R | AGCACCAATCCCTTCACAGA |
| Flt4-F | ACAGAAGCTAGGCCCTACTG |
| Flt4-R | ACCCACATCGAGTCCTTCCT |
| Vegfd-F | CCATCGCTCCACCAGATTTG |
| Vegfd-R | ACACATCACACCCTCTTCGT |
| Agtr1a-F | TGCCATGCCCATAACCATCTG |
| Agtr1a-R | CGTGCTCATTTTCGTAGACAGG |
| Gapdh-F | AACGACCCCTTCATTGACCT |
| Gapdh-R | ATGTTAGTGGGGTCTCGCTC |

**SI-2. Captions of Supplementary Movies 1-2**

**Supplementary Movie 1.** Video of SG and their surrounding microvasculature in WT mice.

**Supplementary Movie 2.** Video of SG and their surrounding microvasculature in *Cthrc1*^-/-^ mice.

**SI-3. The expression levels of SG function genes in mice**


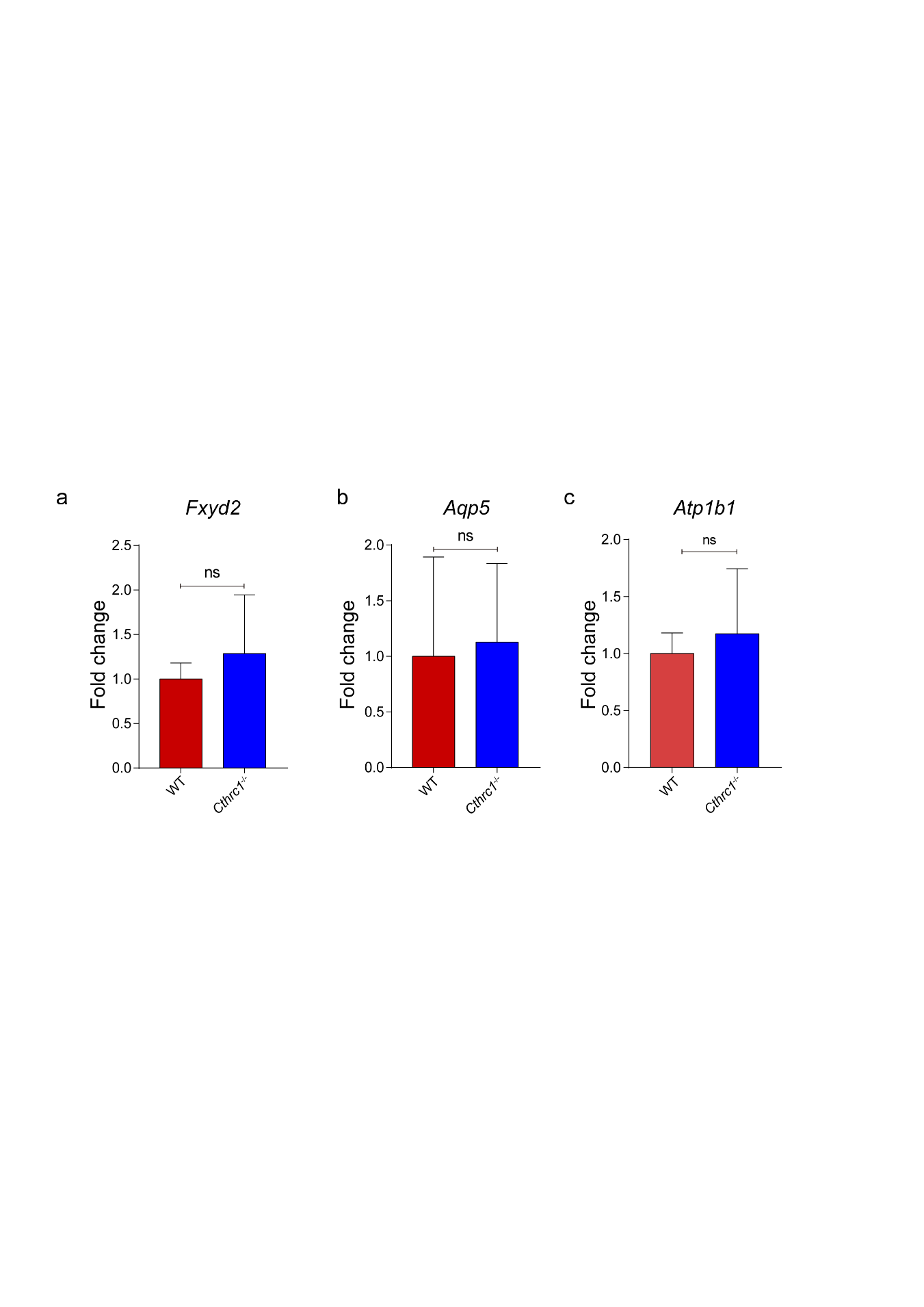


**Figure S1** The relative expression values of *Fxyd2*(**a**)*, Aqp5*(**b**) *and Atp1b1*(**c**) mRNA of SGs of WT and *Cthrc1^-/-^* mice (n=3). Results are presented as the mean ± SD. ns, not significant. *Cthrc1* Collagen triple helix repeat containing-1 gene; *SG* sweat gland, *WT* wild type, *Fxyd2* FXYD domain-containing ion transport regulator 2, *Aqp5* aquaporin 5, *Atp1b1* Na^+^/K^+^ transporting beta 1 polypeptide, *mRNA* messenger RNA, *SD* standard deviation
